# Cross-Sectional Survey of Horse Owners to Assess Their Knowledge and Use of Biosecurity Practices for Equine Infectious Diseases in the United States

**DOI:** 10.3390/ani13223550

**Published:** 2023-11-17

**Authors:** Nathaniel White, Angela Pelzel-McCluskey

**Affiliations:** 1 Equine Disease Communication Center, 4033 Iron Works Pkwy, Lexington, KY 40511, USA; 2 United States Department of Agriculture Animal, Plant Health Inspection Service, Veterinary Services, 2150 Centre Ave., Bldg B, Fort Collins, CO 80526, USA; angela.m.pelzel-mccluskey@usda.gov

**Keywords:** biosecurity, horse owner, horse use, infectious disease, disease risk, survey

## Abstract

**Simple Summary:**

Biosecurity practices are essential to protect the United States horse industry from infectious disease outbreaks. Horse owners’ level of knowledge and awareness of biosecurity are not known. To inform the industry, the current practices need to be understood to determine the kind of education needed to improve biosecurity on owners’ properties or when traveling to horse events. A survey, which consisted of a website-based questionnaire and collection, was distributed to horse owner organizations’ email lists. Evaluation of the results included analysis with a cross-tabulation software to identify significant differences in biosecurity practices associated with horse use and location within the United States. Four areas in the United States were identified (Northeast, Midwest, South and West), and horse use was classified as follows: Pleasure/Trail Riding, Lessons/School, Western Show, English Show, Breeding, Farm/Ranch, Retired, Racing and Driving. In total, 2413 responses were received to the 24-question survey. Differences in biosecurity use and understanding were identified across horse use categories and regions of the country, including differences in the availability of biosecurity plans, risk assessment for horse contact and the use of isolation to mitigate infectious disease. Owners are responsible for medical decision making, and veterinarians are the most trusted resource for medical information. Owners indicated that websites are the preferred way to receive educational information about diseases. There are several biosecurity methods that owners can apply to benefit horse health and welfare. These include temperature monitoring, isolation of new horses at facilities, understanding the risks of horse mingling, knowing the entry requirements such as vaccination and health certificates at events, and having a biosecurity plan for facilities and events where horses come into contact.

**Abstract:**

Horses are transported in the United States more than any other livestock species and co-mingle at various events; therefore, they are considered to be at an increased risk for infectious disease transmission. The fragmented movement of horses combined with numerous sites of co-mingling makes tracing the potential spread of a disease outbreak a necessary part of an infection control plan, both locally and nationally. The cross-movement of personnel with horses and the persistence of endemic diseases make biosecurity implementation an ongoing challenge. Although many of the risks for infection are known, there is limited documentation about the usefulness of prospective control measures. The objective of this survey was to determine horse owners’ understanding and knowledge of biosecurity practices for preventing infectious diseases in the United States. Questions covered owner demographic information, including horse use which was divided into 10 categories as follows: Pleasure/Trail Riding, Lessons/School, Western Show, English Show, Breeding, Farm/Ranch, Retired, Racing, Driving and Other. The survey was distributed by sending requests to a list of horse owner organizations, which then sent emails to their members. The email request described the survey and provided a website link to start the survey. A total of 2413 responses were collected. Analysis of the results included cross-tabulation to identify significant differences in biosecurity knowledge and awareness by horse use. Significant differences by horse use were identified for vaccination, biosecurity planning, use of isolation, disease risk, monitoring for diseases, co-mingling of horses, sanitation, medical decision making and health record requirements for horse events. In summary, the results suggest that most owners are not highly concerned about the risk of disease or the use of biosecurity. There are several biosecurity applications and techniques which can be increased and will benefit horse health and welfare. These include reliance on temperature monitoring, isolation of new horses at facilities, risks of horse mingling, entry requirements such as vaccination and health certificates at events, and an emphasis on having biosecurity plans for facilities and events where horses co-mingle. The information from this study will be used to create tools and information that horse owners and veterinarians can use to implement appropriate biosecurity practices for different types of horse uses and events.

## 1. Introduction

Biosecurity is defined as procedures intended to protect humans or animals against diseases or harmful biological agents. The implementation of biosecurity techniques is the chief way to prevent and respond to infectious diseases in horses. Some aspects of biosecurity and its use were evaluated with the equine National Animal Health Monitoring System (NAHMS) survey in 2015–2016, which examined the biosecurity management of horse operations in 28 states (Biosecurity Assessment of U.S. Equine Operations, USDA–APHIS–VS–CEAH–NAHMS, 2015, https://www.aphis.usda.gov/animal_health/nahms/equine/downloads/equine15/Eq2015_Rept4.pdf (accessed on 1 June 2023). Topics included visitor precaution, vaccination rate and delivery, management of feed and water, control of insects, manure management, and non-resident contact and movement. Health requirements for newly introduced resident equids were also examined.

Surveys to evaluate owner biosecurity practices were completed regarding equine influenza in Australia [1] and visitor protocols in New Zealand [2] and biosecurity practices in Ontario, Canada [3]. Most other studies describe the use of biosecurity for specific disease outbreaks [4]. According to Weese (2014), objective research resulting in recommendations for equine infection control is limited and “*based on basic principles of infectious diseases, common sense, expert opinion and extrapolation from other species*” [5].

Horses are transported more than any other livestock species and co-mingle at various events; therefore, they are considered to be at an increased risk for the transmission of infectious diseases. The fragmented movement of horses combined with numerous sites of co-mingling makes tracing the potential spread of a disease outbreak a necessary part of an infection control plan, both locally and internationally. The cross-movement of personnel with horses and the persistence of infectious diseases make biosecurity implantation an ongoing challenge. Although many of the risks for infection are known, there is limited documentation about the usefulness of prospective control measures [5].

The March 2021 outbreaks of equine herpesvirus myeloencephalopathy in Valencia, Spain, (which caused significant horse loss and curtailed much of the European horse industry, underscores the lack of knowledge about pre-planning for biosecurity at a majority of equine events where horses coming [6,7]. The EHV-1 variant, which was subsequently discovered in Pennsylvania, highlights the possible trans-boundary spread of equine diseases or the potential development of new variants [8]. The spread of EHV in California during the spring of 2022 is an example of the need for biosecurity plans for horse shows and stables (Equine Disease Communication Center, https://www.equinediseasecc.org (accessed on 1 June 2023); California Department of Food and Agriculture, https://www.cdfa.ca.gov/ahfss/Animal_Health/pdfs/Biosecurity_Toolkit_Full_Version.pdf (accessed on 1 June 2023)).

Recent epidemiologic studies detected respiratory viruses EHV-1, EHV-4 and influenza virus in the nasal passage and lymph nodes of horses as well as in the facility environment confirming the potential risk of infection during a horse show and in recently imported horses [9,10,11]. EHV-1 and EHV-4 were also detected from feed bins and water troughs in addition to affected horses, confirming the risk of transmission during an outbreak [12]. Although EHV-4 usually causes mild clinical signs, a 17-week outbreak in Switzerland resulted in disease spread on a breeding farm, predominantly affecting foals. Failure to separate affected from unaffected horses due to insufficient space to isolate affected horses resulted in disease spread [13].

A similar point of care testing using PCR detected salmonella in feces and the environment in veterinary hospitals highlight the need for biosecurity in the hospital environment [14]. The 2019 study for equine influenza outbreak in Great Britain concluded the lack of vaccination, effect of new horses arriving at an affected premises and limited implementation of biosecurity measures as reasons for the epidemic [15].

Although veterinarians are trained in management of infectious diseases, horse owners appear to lack the knowledge needed to prepare for and manage a disease outbreak. Although humans have learned about biosecurity through the need to manage COVID-19 during 2020–2023, it is not clear that horse owners have gained an increased understanding of biosecurity or risk assessment which is needed for disease prevention. To be able to prevent and control diseases, a basic understanding of the concepts of biosecurity and infection control is necessary [5].

Previous horse owner surveys have been completed in Canada and the United Kingdom [3,16]. These included a survey to determine the risk of infectious disease dissemination based on horse location and movement. A lack of acceptance of biosecurity practice was identified on Australian Thoroughbred breeding farms in spite of the availability of training [17]. Recommendations for owner and veterinarian applications of biosecurity for horses is available from the USDA, state governments, universities and horse owner publications, but there are few reports about owner use or successes in preventing disease (Equine Disease Communication Center, https://www.equinediseasecc.org (accessed on 1 June 2023; California Department of Food and Agriculture, https://www.cdfa.ca.gov/ahfss/Animal_Health/pdfs/Biosecurity_Toolkit_Full_Version.pdf (accessed on 1 June 2023); Colorado State University, Animal Biosecurity, https://animalbiosecurity.colostate.edu/horse-overview-owner/ (accessed on 1 June 2023)).

The objective of this survey was to determine horse owners’ understanding and knowledge of biosecurity strategies for preventing infectious diseases in the United States. Once identified, the results will be used to create tools and information that horse owners and veterinarians can use to implement appropriate biosecurity measures for different types of horse uses and events.

## 2. Materials and Methods

Survey questions were developed by infectious disease experts from the American Association of Equine Practitioners’ (AAEP) Infectious Disease Committee. The number of questions (24) was based on what was considered to achieve optimal participation while still obtaining sufficient information to fulfill the survey objective. The survey was pretested by 8 horse owners to ensure the clarity of the questions and determine the time required to complete the survey.

Six demographic questions were included to determine ownership, location by state/province, facility type, horse use (discipline) and medical decision making. The remaining questions (questions 7–24) were used to determine the horse owner’s response to vaccine use, disease risk due to horse and human contact, knowledge of biosecurity techniques, and preferences regarding information presentation. Horse use was divided into 10 categories, and owners were able select more than one use for their horse(s).

- Pleasure/Trail Riding;
- Lessons/School;
- Western Show;
- English Show;
- Breeding;
- Farm/Ranch;
- Retired;
- Racing;
- Driving;
- Other.

These questions were placed on a website created for the participants, and the responses were collected and analyzed by a market research company (The Matrix Group, Lexington, KY, www.tmgresearch.com (accessed on 1 June 2023)). The survey was distributed by sending requests to a list of horse owner organizations, which then sent emails to their member lists (Table 1). The email request described the survey and included a website link to enable survey participation.

Statistical analysis was completed with a cross-tabulation software (Wincross statistical reference, The Analytical Group, Inc., 15300 N. 90th Street, Scottsdale, AZ 85260, USA, www.analyticalgroup.com (accessed on 1 June 2023)), which used a Z-test to identify significant differences between the percentages in ten use categories for each response within each question. A Z-value greater than 2 standard deviations (95%) above the mean was accepted as significantly different. Only responses that were considered significantly different in a use category compared to all uses were reported. Comparing each use to the other uses independently for each response potentially made more than one use significantly different from the remaining uses possible.

## 3. Results

There were 2413 responses to the horse owner survey. Responses were submitted from 49 states. States were assigned to one of four regions in the United States:

- Northeast—Connecticut (CT), Maine (ME), Massachusetts (MA), New Hampshire (NH), Rhode Island (RI), Vermont (VT), New Jersey (NJ), New York (NY) and Pennsylvania (PA);
- South—Alabama (AL), Kentucky (KY), Mississippi (MS), Tennessee (TN), Delaware (DE), Florida (FL), Georgia (GA), Maryland (MD), North Carolina (NC), South Carolina (SC), Virginia (VA), West Virginia (WV), Arkansas (AR), Louisiana (LA), Oklahoma (OK) and Texas (TX);
- Midwest—Illinois (IL0, Indiana (IN), Michigan (MI), Ohio (OH), Wisconsin (WI), Iowa (IA), Kansas (KS), Missouri (MO), Nebraska (NE), North Dakota (ND) and South Dakota (SD);
- West—Arizona (AZ), Colorado (CO), Idaho (ID), Montana (MT), Nevada (NV), New Mexico (NM), Utah (UT), Wyoming (WY), Alaska (AK), California (CA), Oregon (OR) and Washington (WA).

The proportion of responses from horse owners was similar to the estimated population of horses in each state (Supplementary Table S1) (American Horse Publications, https://www.americanhorsepubs.org/2021-equine-survey/ (accessed on 1 June 2023)).

Responses to each question are presented in Table 2 and Table 3. This includes significant differences in the response for each question based on horse use. Because some of the questions requested more than one response, such as “select all that apply”, the percentages reported for those questions are potentially cumulative.

Of the 2413 horse owners responding to the survey, 76.3% owned 1–5 horses. The owners’ disciplines varied by region in the U.S., with English Show predominating in the Northeast, Breeding being significantly higher in the South and Midwest, and no significant difference found in the Western U.S. A majority of horses are used for Pleasure/Trail Riding (55.5%), followed by English Show (40.2%) (Table 2). Owners are the primary decision maker for their horse’s medical care (91.6%) compared to trainers and veterinarians, and when selecting the top 3 sources for infectious disease information, veterinarians ranked highest (98.8%). Owners rely on veterinarians (93.2%) to determine the appropriate vaccine for their horses, with 78.6% of vaccines administered by veterinarians.

**Figure 1:**
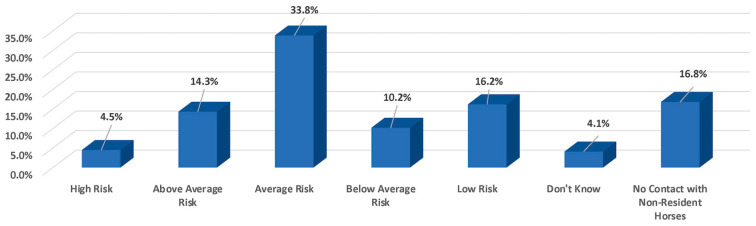
Perceived risk of disease transmission due to contact with non-resident horses.

Sixty percent (59.9%) of owners keep their horses on their property vs. at boarding facilities (39.8%), and seventy-five percent of owners indicated that their horse came into contact with non-resident horses on one or more days during the year, with this being significantly higher in the Western Show discipline (*p* ≤ 0.05). However, 60.2% of owners consider contact with non-resident horses to be of average to low risk for disease transmission (Figure 1).

Fifty-four percent (54.2%) of facilities have a plan for the isolation of horses with an infectious disease. Similarly, in a separate question it was found that 54.4% of facilities require separate housing for new horses moved to the facilities, which is significantly higher for Breeding, Ranch/Farm and Racing/Other facilities (*p* ≤ 0.05).

**Figure 2:**
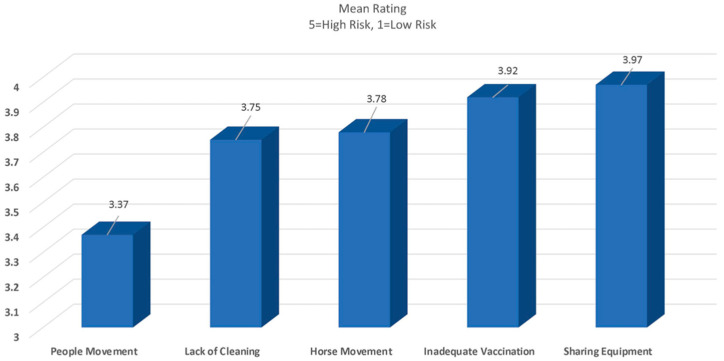
Owner perception of the level of risk for disease transmission due to movement of people, lack of cleaning, horse movement, inadequate ventilation and sharing of equipment.

The level of risk for specific disease transmission factors ranked by owners is presented in Figure 2.

**Figure 3:**
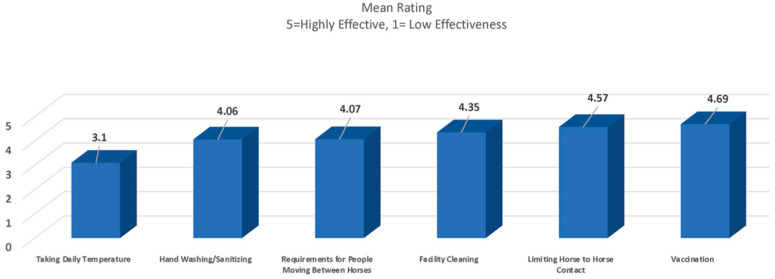
Owner ranking of effectiveness of biosecurity practices for common respiratory infections.

When evaluating the effectiveness of biosecurity techniques for respiratory diseases (such as influenza, strangles and equine herpesvirus), owners ranked vaccination as the most effective, whereas taking a daily temperature ranked the lowest (Figure 3).

**Figure 4:**
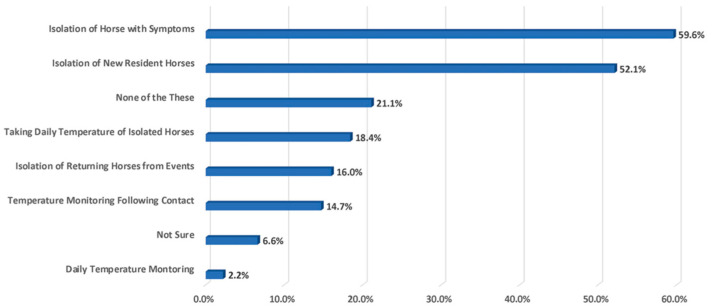
Ranking of biosecurity practices used by owners or facilities where horses reside.

Regarding biosecurity practices at facilities, the isolation of sick animals ranks the highest (59.6%), while taking the temperature ranks the lowest (2.5%) (Figure 4).

**Figure 5:**
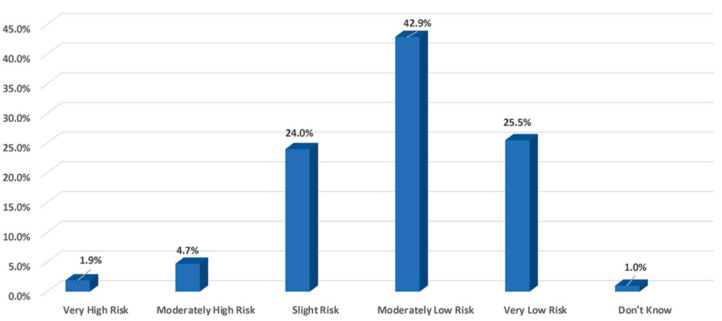
Perceived risk of contracting a disease during co-mingling at horse events.

Ninety two percent (92.4%) of owners consider co-mingling of horses at events (i.e., showing, racing, and trail rides) to be a slight to very low risk for contracting an infectious disease (Figure 5).

**Figure 6:**
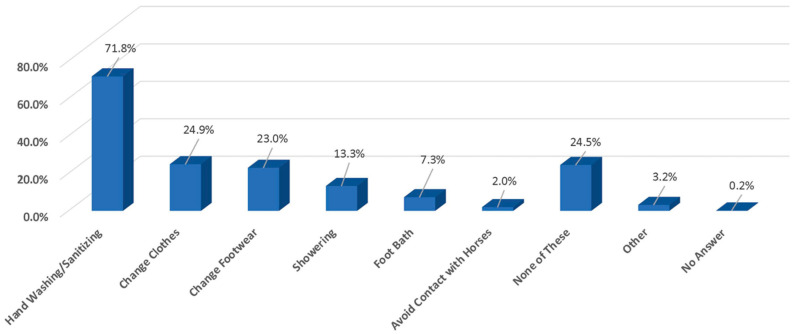
Owner selection of the most effective precautions after an owner and/or an owner’s horse were in contact with non-resident horses.

Owners selected hand sanitation as the most effective precaution after contact with a non-resident horse, and this was selected nearly three times more than any of the other listed precautions (Figure 6).

**Figure 7:**
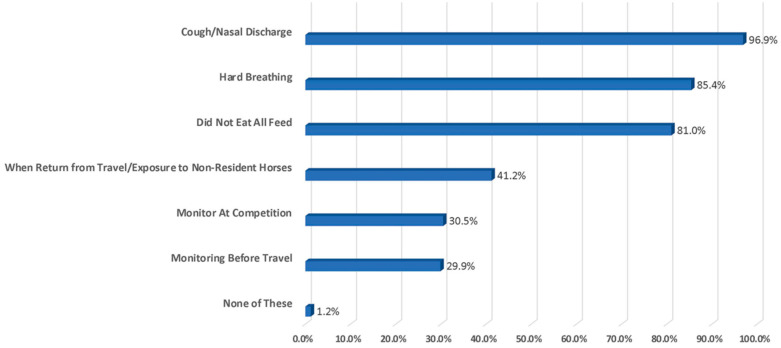
Selection of items that prompt owners to take a horse’s temperature.

Taking the horse’s temperature was highly ranked for horses showing signs of a respiratory disease (96.9%), while taking their temperature prior to travel for an event was not selected as often (29.5%) (Figure 7).

**Figure 8:**
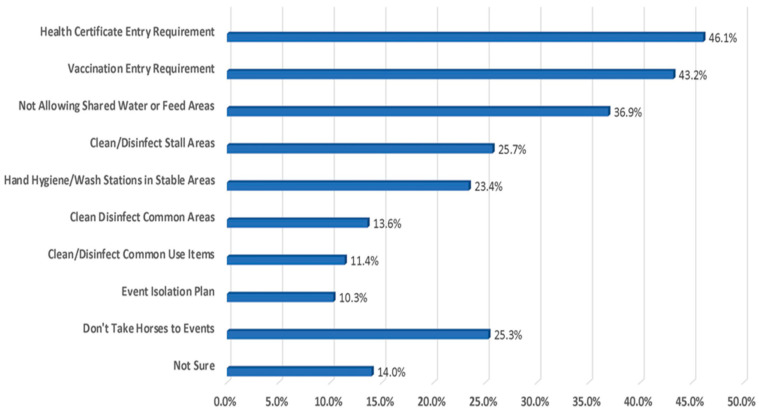
Provisions most commonly in place at facilities where horses are taken for competitions or events.

Owners consider the most common biosecurity provisions in place at facilities where horses are taken for competitions or events to be requiring health certificates and vaccinations for entry, while having an event isolation plan came in as the lowest at 10.2% (Figure 8).

**Figure 9:**
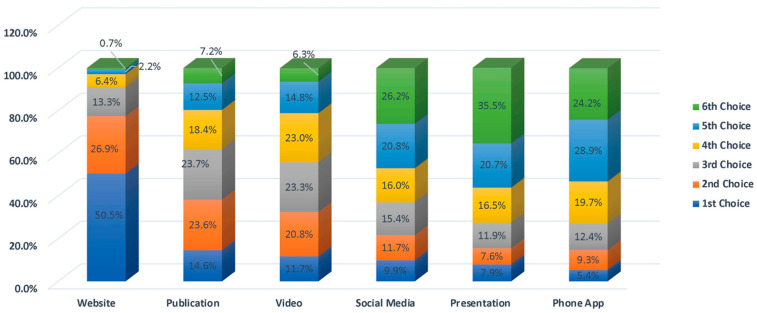
Horse owner preference for how biosecurity information is best presented.

When owners were asked to identify their first choice for obtaining information about biosecurity, 50.5% selected a website, followed by a publication at 14.6% and a video at 11.7% (Figure 9).

## 4. Discussion

This is the second national-level survey to examine the U.S. horse industry’s use and perception of biosecurity strategies. The 2015 NAHMS survey investigated owners’ responses in 28 states and involved detailed information about horse demographics including location, breed, use, age, gender, health records, husbandry (including environmental biosecurity), medical decisions, medical care, parasite control, vaccinations, knowledge of diseases and biosecurity, as well as contact with non-resident horses (Baseline Reference of Equine Health and Management in the United States, USDA–APHIS–VS–CEAH–NAHMS, 2015, https://www.aphis.usda.gov/animal_health/nahms/equine/downloads/equine15/Eq2015_Rept1.pdf (accessed on 1 June 2023)). A portion of the 2015 NAHMS study was a biosecurity assessment (Biosecurity Assessment of U.S. Equine Operations, USDA–APHIS–VS–CEAH–NAHMS, 2015, https://www.aphis.usda.gov/animal_health/nahms/equine/downloads/equine15/Eq2015_Rept4.pdf (accessed on 1 June 2023)). Facility cleanliness, fly control, manure storage, health records, isolation capabilities and personal protective equipment (PPE) availability addressed in the NAHMS study were not specifically assessed in our study. In comparison, we identified location by state, predominate use and potential horse contact, while moving off the primary residence, and co-mingling at events. Owners’ opinions about the need for biosecurity and what factors or techniques were considered important were also surveyed.

Because the distribution of owner responses is strongly correlated with the estimated number of horses in each state, the survey is considered representative of the horse industry in the United States (American Horse Publications, https://www.americanhorsepubs.org/2021-equine-survey/ (accessed on 20 June 2023)). The breakdown for use disciplines is similar to the results of the 2015 NAHMS study (Baseline Reference of Equine Health and Management in the United States, USDA–APHIS–VS–CEAH–NAHMS, 2015, https://www.aphis.usda.gov/animal_health/nahms/equine/downloads/equine15/Eq2015_Rept1.pdf (accessed 1 June 2023)), with Pleasure/Trail Riding identified as the predominate use (55.5%). However, our results do not account for the multiple uses possible for each horse.

The significant differences in responses associated with use suggest differing owner management strategies, economic means or horse-owning cultures [5]. Examples include the significant increased use of “at-risk vaccines” in English Show horses and significantly more horses used for Pleasure/Trail Riding, Lessons/School and English Show vaccinated by a veterinarian compared to other uses (AAEP Vaccination guidelines, https://aaep.org/guidelines/vaccination-guidelines/core-vaccination-guidelines; (accessed on 26 October 2023). This suggests an opportunity to educate owners about the benefits of vaccination, particularly where there are lower vaccination rates in some disciplines.

Although 92.4% of owners perceive that the risk of disease transmission during the co-mingling of horses is slight to very low, isolation of a new horse introduced to a facility or horses suspected of having a disease, limiting horse contact, and vaccination were considered the most effective ways to limit disease spread. Similar to the NAHMS study, the percentage of veterinarians who administer the vaccines is highest for horses in a boarding stable, while it is lowest in farm/ranch horses (Biosecurity Assessment of U.S. Equine Operations, USDA–APHIS–VS–CEAH–NAHMS, 2015; https://www.aphis.usda.gov/animal_health/nahms/equine/downloads/equine15/Eq2015_Rept4.pdf (accessed on 1 June 2023); Baseline Reference of Equine Health and Management in the United States, USDA–APHIS–VS–CEAH–NAHMS, 2015, https://www.aphis.usda.gov/animal_health/nahms/equine/downloads/equine15/Eq2015_Rept1.pdf (accessed on 1 June 2023)). Taking temperatures after horse-to-horse contact or daily temperature monitoring are not ranked as high, emphasizing the need for education about the value of taking a horse’s temperature as part of biosecurity [2].

Although 91.6% of owners make the medical decisions for their horses, 88.9% rely on veterinarians for medical and biosecurity information, which is more than the percentage reported in the 2015 NAHMS study (78.8%) (Biosecurity Assessment of U.S. Equine Operations, USDA–APHIS–VS–CEAH–NAHMS, 2015, https://www.aphis.usda.gov/animal_health/nahms/equine/downloads/equine15/Eq2015_Rept4.pdf (accessed on 1 June 2023)). Seventy nine percent (78.6%) of owners have their horses vaccinated by a veterinarian, which was more than the percentage reported in the larger American Horse Publications 2021 survey (65.4%) (American Horse Publications, https://www.americanhorsepubs.org/2021-equine-survey/ (accessed on 1 June 2023)). This activity gives veterinarians access to the owners and can be used as an opportunity to help improve the owners’ awareness of the benefits of biosecurity.

Overall, 54.2% of facilities in our survey have a biosecurity plan, leaving 45% with no plan (31.4%) or no knowledge of a plan (14.3%), which is similar to the NAHMS and Ontario study [3]. Facilities for lessons and farms/ranches have the highest percentage of plans. These numbers vary in the NAHMS study, with breeding farms having the highest percentage and farms or ranches having the lowest percentage (Biosecurity Assessment of U.S. Equine Operations, USDA–APHIS–VS–CEAH–NAHMS, 2015, https://www.aphis.usda.gov/animal_health/nahms/equine/downloads/equine15/Eq2015_Rept4.pdf (accessed on 1 June 2023)). Owners reported that 54.4% of facilities have a requirement for the separation of new horses at facilities, which is lower than that found in the NAHMS study at 64.8 to 77.5%, depending on the type of separation used. The isolation of new horses is practiced in 52.1% of facilities, whereas 59.6% isolated horses with symptoms of an infectious disease. During a veterinarian biosecurity survey completed at the same time as the owner survey, the most frequent risk factor for infectious diseases was a lack of isolation for new horse arrivals (Equine Disease Communication Center, veterinarian survey for biosecurity, 2023 (unpublished)).

Hand washing/sanitation is the most owner-reported precaution for preventing disease after contact with a non-resident horse (71.8%), which is nearly three times more than changing clothes, changing footwear or other techniques (Table 3) (Figure 6). Hand washing/sanitation was considered adequate in 41.7% of all operations in the NAHMS study, but there was no information about how often it was used (Biosecurity Assessment of U.S. Equine Operations, USDA–APHIS–VS–CEAH–NAHMS, 2015, https://www.aphis.usda.gov/animal_health/nahms/equine/downloads/equine15/Eq2015_Rept4.pdf (accessed on 1 June 2023)).

In the veterinarian survey (Equine Disease Communication Center, veterinarian survey for biosecurity, 2023 (unpublished)), the assessment of facility biosecurity performed by veterinarians was limited, as indicated by just 6.2% of respondents. When veterinarians were asked why they did not offer to make biosecurity plans for owners, most said clients do not want to pay for the service or that the practice does not provide that service (Equine Disease Communication Center, veterinarian survey for biosecurity, 2023 (unpublished)).

Success in limiting infection spread has been reported for previous techniques used at the Olympic games, and recent reports of preventing the spread of equine herpesvirus at events, racetracks and farms suggests that these plans are effective [5]. However, the lack of information about transmission in various environments makes it difficult to know if specific biosecurity techniques are effective. Several biosecurity documents provide specific recommendations and resources for setting up biosecurity at an event (Equine Disease Communication Center, https://www.equinediseasecc.org (accessed on 1 June 2023); California Department of Food and Agriculture, https://www.cdfa.ca.gov/ahfss/Animal_Health/pdfs/Biosecurity_Toolkit_Full_Version.pdf (accessed on 1 June 2023)). Common features to be considered include minimizing horse-to-horse contact, vaccine requirements, stall sanitation, non-shared drinking water, etc. [9]. Recommendations are also described including health requirements for entry, isolation of sick horses, temperature monitoring, limiting horse-to-horse and human-to-horse contact, vector control, and record keeping.

There are only a few surveys reporting on disease transmission in horses that travel. An owner survey in Great Britain found most traveling horses were vaccinated, and the greatest risk for disease introduction may be from a small group of individuals who import or travel internationally with their horses [16]. Unfortunately, the risk of horses becoming infected during travel has not been measured.

Breaks in biosecurity or lack of use have been reported to be responsible for disease outbreaks of salmonella, influenza and equine herpesvirus [2,7,8,9,18]. There is no documented effectiveness of applying biosecurity techniques to normal equine populations, but the lack of having a biosecurity plan is associated with the inability to contain an outbreak [5].

A survey taken after an equine influenza outbreak in Australia indicated that low biosecurity compliance occurred most often when there was no commercial involvement with horses [1]. Other than our study and the NAHMS study (Biosecurity Assessment of U.S. Equine Operations, USDA–APHIS–VS–CEAH–NAHMS, 2015, https://www.aphis.usda.gov/animal_health/nahms/equine/downloads/equine15/Eq2015_Rept4.pdf (accessed on 1 June 2023)), there is little information about what owners actually use to prevent infectious disease. Determining the effectiveness of specific techniques to prevent disease transmission will require long-term on-site comparisons of similar facilities and populations that have different levels of biosecurity.

The limitations of our survey included a low response rate, considering the large number of email solicitations sent from numerous sources (Table 1). Email provides a faster response speed and is more effective than postal surveys, but the overall quality has not been shown to be different [19]. Owner online surveys, which are not standardized, are susceptible to self-selection and sampling bias. Sampling bias, which can be decreased with a standardized and validated survey tool, can prevent comparisons with other studies [19]. Our study was not validated and would require larger and repeated surveys to achieve validation. In this survey, there was no way to target the audience or stimulate participation other than by having the different organizations request that members take the survey. The use of the email addresses by the organization could create bias as the criteria for selection is not known.

The proportion of owners was similar to the estimated number of horse in each state. However, the survey was not a census, and therefore, we cannot be sure that the survey represents a relationship of the actual number of horses to owners or that the survey accurately is reflective of the horse industry (Supplemental Table S1).

Several important topic areas surveyed in the NAHMS study (Biosecurity Assessment of U.S. Equine Operations, USDA–APHIS–VS–CEAH–NAHMS, 2015, https://www.aphis.usda.gov/animal_health/nahms/equine/downloads/equine15/Eq2015_Rept4.pdf (accessed on 1 June 2023)), including vectors, manure and water management, and facility sanitation, were not within the scope of this project. These are important areas where information is already available for horse owners (California Department of Food and Agriculture, https://www.cdfa.ca.gov/ahfss/Animal_Health/pdfs/Biosecurity_Toolkit_Full_Version.pdf (accessed on 1 June 2023); Colorado State University, Animal Biosecurity, https://animalbiosecurity.colostate.edu/horse-overview-owner/) (accessed on 1 June 2023)).

## 5. Conclusions

The goal of this survey was to identify horse owners’ understanding and use of biosecurity techniques. Based on the responses, there are several biosecurity applications and techniques which can be increased and will benefit horses and the horse industry. Specific topics include reliance on temperature monitoring, isolation of new horses at facilities, risks of horse mingling, entry requirements such as vaccination and health certificates at events, and an emphasis on having biosecurity plans for facilities and events where horses co-mingle. Educating owners about assessing risk in different environments is needed to show why specific biosecurity actions can decrease infectious disease prevalence. Furthermore, because significant differences in biosecurity use were identified for different disciplines and horse uses, biosecurity information can be targeted by discipline and breed organization. Examples from our study include establishing biosecurity plans at Pleasure/Trail Riding facilities and encouraging an isolation requirement for new horses coming to English Show facilities. Veterinarians should find the results helpful when helping horse owners to establish biosecurity plans for the management of their horses. Coordinating biosecurity recommendations on industry websites and through the publication of information in horse industry media will be the best way to reach and educate horse owners.

## Figures and Tables

**Table 1:** Email lists used to distribute the horse owner survey. Two requests were sent to the email lists 6 weeks apart. Two survey requests were posted on the EDCC Facebook page.

American Quarter Horse Association—60,000 email addresses
United States Equestrian Federation—36,000 email addresses
American Horse Council—800 email addresses
Equine Disease Communication Center subscribers—8600 email addresses
Equine Disease Communication Center Facebook—18,000 followers

**Table 2:** Survey questions (1–6) were used to identify horse and horse owner demographics, including significant differences based on horse use. Percentages of responses for horse use which were significantly higher than *p* < 0.05 are in parentheses.

1. How many horses do you own or lease?	
○ 45.2% owned 1–2 horses ○ 31.1% owned 3–5 horses ○ 10.9% owned 6–9 ○ 12.6% owned >10	
2. Indicate the state or Canadian province where your horse(s) reside for more than 2 months of the year.	
○ *South region* (*Breeding*) ○ *Northeast region* (*English Show*) ○ *Midwest region* ○ *West region* ○ *Canada* (*English Show*)	
2A. Indicate the other state or Canadian province where your horse(s) reside for more than 2 months of the year.	
○ *South region* (*Breeding and Racing*) ○ *Northeast region* (*Racing*) ○ *Midwest region* (*Breeding*) ○ *West region* ○ *Canada*	
3. What is/are the primary use(s) of the horse(s) you own? (SELECT ALL THAT APPLY) (The percentage of responses may include more than one use).	
Pleasure/Trail Riding	55.5%
Lessons/School	14.3%
Western Show	27.6%
English Show	40.2%
Breeding	18.8%
Ranch/Farm	11.3%
Retired	27.4%
Racing	2.%
Driving	1.7%
Other	2.6%
4. Who is the primary decision maker for your horse’s medical care?	
○ *Myself* (*owner*): 91.6% (*Pleasure/Trail, Farm/Ranch and Retired*) ○ *Trainer*: 3.0% (*Lessons/School and English Show*) ○ *Veterinarian*: 5.0% ○ *Boarding facility*: 0.3%	
5. Where do you keep your horse(s)?	
○ *On my property*: 59.6% (*Breeding and Farm/Ranch*) ○ *At a boarding facility*: 39.8% (*Lessons/School and English show*) ○ *Commercial facility*: 0.7% (*Racing*)	
6. Indicate the primary resources where you get information on prevention of infectious diseases for your horse(s). SELECT UP TO 3 USED MOST OFTEN.	
○ *Veterinarian*: 88.9% ○ *Other horse owners*: 23.4% ○ *EDCC*: 23.2% ○ Social media: 19.3% ○ *Trainer*: 18.6% (*English Show*) ○ *AAEP*: 18.1% (*Retired*) ○ *Horse owner magazine*: 16.3% ○ *Google search/internet*: 16.0% ○ *State animal state official*: 9.8% ○ *Farrier*: 8.5% ○ *USDA*: 8.3% ○ National equestrian association: 7.0% ○ *University/Extension*: 1.5% ○ *Journal/Publications*: 0.7% ○ Other: 2.3%	

**Table 3:** Survey questions (7–24) identify the knowledge of biosecurity horse owners to prevent infectious diseases, including significant differences based on horse use (Pleasure/Trail Riding; Lessons/School; Western Show: English Show; Breeding; Farm/Ranch; Retired; Racing/Other). Percentages of responses for horse use which were significantly higher (*p* < 0.05) are in parentheses.

7. Have you asked your veterinarian which vaccines are appropriate for your horse(s) based on their risk for contracting an infectious disease?
○ *Yes*: 93.2% (*Lessons/School*) ○ *No*: 6.8%
8. Which of the following vaccines do your horses receive each year? SELECT ALL THAT APPLY.
○ *Core vaccines*: 87.5% (*Pleasure/Trail, Lessons/School and English Show*) (https://aaep.org/guidelines/vaccination-guidelines/core-vaccination-guidelines; (accessed on 26 October 2023) ○ *Risk-based vaccines*: 77.0% (*English Show*); (https://aaep.org/guidelines/vaccination-guidelines/core-vaccination-guidelines (accessed on 26 October 2023) ○ Vaccinated but do not know which vaccines: 3.1% ○ *Not vaccinated*: 2.2% (*Breeding, Farm/Ranch and Retired*)
9. Who administers vaccines to your horses?
○ *My veterinarian*: 78.6% (*Pleasure/Trail Riding, Lessons/School and English Show*) ○ *Myself* (*responder*): 21.8% (*Breeding, Farm/Ranch and Western Show*) ○ I do not vaccinate my horses: 0.8% ○ *Boarding facility*: 0.7% ○ *My trainer*: 0.6% ○ *Another horse owner*: 0.1%
10. Other than through vaccinations, have you discussed how to prevent your horses from getting an infectious disease with your veterinarian?
○ *Yes*: 66.8% (*Lessons/School*) ○ *No*: 33.2%
11. Is there a plan for preventing infectious diseases at the facility where you keep your horses?
○ *Yes*: 54.2% (*Lessons/School*) ○ *No*: 31.4% (*Pleasure/Trail Riding*) ○ *Don’t know*: 14.3% (*English Show*)
12. Is there a temporary isolation requirement for new horses moved to the facility where your horse(s) reside?
○ Yes: 54.4% (*Breeding, Farm/Ranch and Racing*) ○ No: 41.1% (*English Show*) ○ *Don’t know*: 4.5%
13. On average, how many days per year does your horse(s) have contact with non-resident horses?
○ *Zero*: 25.1% (*Pleasure/Trail and Retired*) ○ 10 *or fewer*: 27.1% ○ 11 *to* 29: 18.9% ○ 30 *or more*: 27.5% (*Western Show*) ○ *Other*: 1.5%
14. How often do you take your horse’s temperature prior to participating in an event or group activity?
○ *Always*: 10.2% (*English Show*) ○ *Sometimes*: 19.3% (*English Show*) ○ *Rarely*: 24.5% (*Western Show*) ○ *Never*: 27.7% (*Western Show*) ○ *I do not have a thermometer*: 1.3% ○ *No participation in events or group activity*: 17.1% (*Pleasure/Trail, Retired and Racing*)
15. If your horse has contact with horses that normally do not reside at your facility (non-resident horse), what is your perception of the risk for your horse acquiring a respiratory infection?
○ *High risk*: 4.5% (*Breeding*) ○ *Above average risk*: 14.3% ○ *Average risk*: 33.8% ○ *Below average risk*: 10.2% ○ *Low risk*: 16.2% ○ *Don’t know/Not sure*: 4.1% ○ *No non-resident contact*: 16.8% (*Pleasure/Trail and Retired*)
16. On a 5 to 1 scale, with 5 being high risk and 1 being low risk, please rate the level of risk of the following factors for infectious disease transmission or disease introduction.
○ *Lack of cleaning or disinfection*: 3.75/5 ○ *Horse movement on and off property*: 3.78/5 (*English Show and Retired*) ○ *Sharing of equipment* (*tack, grooming, water buckets, stall cleaning equipment, wipe rags, etc.*): 3.97/5 ○ *People movement at facility*: 3.37/5 ○ *Inadequate vaccination* 3.9/5 (*English Show*)
17. On a 5 to 1 scale, with 5 being highly effective and 1 having low effectiveness, rate each of the following biosecurity techniques on how effective you think each is in preventing equine respiratory infections (such as influenza, strangles, and herpesvirus) in your horse(s).
○ *Taking daily temp*: 3.1/5 (*English Show*) ○ *Hand washing/sanitizing*: 4.06/5 ○ *Facility cleaning*: 4.35/5 ○ *Requirements for people moving between horses*: 4.07/5 ○ *Vaccination*: 4.69/5 ○ *Limited horse contact*: 4.57/5 (*English Show and Retired*)
18. Which of the following biosecurity practices do you or does your facility management routinely use where your horse resides? (SELECT ALL THAT APPLY)
○ *Isolating any horse showing respiratory signs of a temp* >*101.5 degrees*: 59.6% ○ *Isolating new resident horses*: 59.6% (*Breeding and Farm/Ranch*) ○ *None of the above*: 21.5% (*Pleasure/Trail*) ○ *Taking daily temp of isolated horses*: 18.4% ○ *Isolating resident horses returning from event*: 16.0% (*Breeding and Farm/Ranch*) ○ *Temp monitoring follow contact with non-resident horses*: 14.7% ○ *Daily temp monitoring*: 2.2% (Racing) ○ *Not sure*: 6.6%
19. After you and your horse are in contact with non-resident horses do you take any of the following precautions before visiting your own horse? (SELECT ALL THAT APPLY)
○ *Hand washing/sanitizing*: 71.8% ○ *Change of clothes*: 24.9% ○ *Change of footwear*: 23.0% ○ *Showering*: 13.3% ○ *Foot bath*: 7.3% ○ *Avoid contact with other horses*: 2.0% ○ *Spray/Wipe with disinfectant*: 0.6% ○ *Sanitize equipment*: 0.5% ○ *Other*: 2.2% ○ *No answer*: 0.2%
20. Which of the following would prompt you to take your horse’s temperature? (SELECT ALL THAT APPLY)
○ *Cough and/or nasal discharge*: 96.9% ○ *Breathing hard*: 85.4% ○ *Did not eat all feed*: 81% ○ *Monitoring when returning from travel and/or exposure to non-resident horses*: 41.2% (*English Show*) ○ *Monitoring at equine competition*: 30.5%: (*English Show*) ○ *Monitoring before travel*: 29.9% (*English Show*) ○ *None of the above*: 1.2%
21. On a 5 to 1 scale, with 5 being very high risk and 1 being very low risk, what is your perception of the level of risk for horses that co-mingle with other non-resident horses during events such as showing, racing, group trail ride or other competitions?
○ Very high risk: 1.9% ○ *Moderately high risk*: 4.7% ○ *Slight risk*: 24.0% ○ *Moderately low risk*: 42.9% ○ *Very low risk*: 25.5% ○ *Don’t know/not sure*: 1.0%
22. On average, which of the following biosecurity provisions are in place at the facilities where you take your horse for competitions or events? (SELECT ALL THAT APPLY)
○ *Health certificate entry requirement*: 46.1% ○ *Vaccination entry requirement*: 43.2% (*Lessons/School and English show*) ○ *Do not allow shared water or feed areas*: 36.9% (*Lessons/School, Western Show and English Show*) ○ *Thoroughly clean and disinfect the stall area between occupants*: 25.7% ○ *I do not take my horses to events*: 25.3% (*Pleasure/Trail Riding, Retired*) ○ *Provide hand hygiene or hand washing stations in the stabling area*: 23.5% (*English Show*) ○ *Clean and disinfect common areas such as wash rack, tie rack on a daily or more frequent basis*: 13.6% ○ *Clean and disinfect common areas such as hoses that are used to fill buckets*: 11.4% ○ *There is an event isolation plan*: 10.3% (*English Show*) ○ *Not Sure*: 14.0% (*Western Show*)
23. Among the following individuals that come in contact with your horse, which do you believe increases the risk to your horse(s) of contracting an infectious disease? (SELECT ALL THAT APPLY)
○ *Veterinary dentist*: 34.1% (*Retired*) ○ *Farrier*: 47.4% ○ *Resident trainer*: 8.4% ○ *Veterinarian*: 36.9% ○ *Feed supplier*: 8.5% ○ *Visiting trainers*: 22.7% (*Lessons/School and English Show*) ○ *Visiting owners*: 44.8% ○ *Traveling groomers/braiders/saddle fitters/massage therapists/lay dentists*: 36.8% (*English Show*) ○ *Transporter/Hauler*: 38.6%
24. When you are looking for information about biosecurity, what is your order of preference for having information presented? (Rank 6 choices)
○ Video: 11.7% ○ *Website*: 50.5% ○ *Publication*: 14.6% ○ *Phone App*: 5.4% ○ *Social Media*: 9.9% ○ *Presentation*: 7.9% (*Farm/Ranch*)

## Data Availability

The data presented in this study are available with approval from the corresponding author.

## Supplementary Materials

The following supporting information can be downloaded at: https://www.mdpi.com/article/10.3390/ani13223550/s1, Table S1: Estimated number of horses in each state (American Horse Publications, https://www.americanhorsepubs.org/2021-equine-survey/ (accessed 1 June 2023)), and the number of horse owner responses. A Pearson correlation coefficient of r = 0.846 suggests a strong linear correlation between the horse population and the number of survey responses from each state (r = 0.84692717).
