# Supplementary material for: Cross-Sectional Survey of Horse Owners to Assess Their Knowledge and Use of Biosecurity Practices for Equine Infectious Diseases in the United States
Source: Animals (Basel). 2023 Nov 17;13(22):3550. doi: 10.3390/ani13223550 (PMC10668770; doi:10.3390/ani13223550)
Supplement: Supplementary file 1 [file animals-13-03550-s001.zip › animals-2647222-supplementary.pdf]

Table S1. Estimated number of horses in each state (American Horse Publications, https://www.americanhorsepubs.org/2021-equine-survey/ (accessed 1 June 2023)), and the number of horse owner responses. A Pearson correlation coefficient of r = 0.846 suggests a strong linear correlation between the horse population and the number of survey responses from each state (r = 0.84692717).

State	Horse Population	Responses by State
CN	40,400	22
ME	28,600	13
MA	30,000	29
NH	11,700	12
RI	2600	3
VT	19,800	12
NJ	59,400	26
NY	154,000	80
PA	223,600	74
IL	150,000	52
IN	173,000	45
MI	190,000	101
OH	256,000	63
WI	150,300	42
IA	44,000	29
KS	139,000	30
MN	146,000	78
MO	230,000	50
NE	117,000	25
ND	47,000	14
SD	96,500	11
AL	114,300	22
KY	238,000	59
MS	78,400	16
TN	165,800	30
DE	8000	1
FL	387,000	105
GA	133,000	31
MD	101,500	101
NC	205,200	66
SC	73,600	39
VA	183,600	81
WV	69,800	10
AR	70,000	13
LA	120,500	41
OK	253,000	39
TX	767,000	172
AZ	142,000	52
CO	265,000	87
ID	12,500	30
MT	104,900	26

NV	41,200	5
NM	113,500	30
UT	96,400	15
WY	78,600	17
AK	9100	4
CA	535,000	243
OR	135,200	59
WA	197,200	104
OTHER		15
Total in US	7,008,200	2413

Key to states initials: Connecticut (CT), Maine (ME), Massachusetts (MA), New Hampshire (NH), Rhode Island (RI), Vermont (VT), New Jersey (NJ), New York (NY), Pennsylvania (PA), Alabama (AL), Kentucky (KY), Mississippi (MS), Tennessee (TN), Delaware (DE), Florida (FL), Georgia (GA), Maryland (MD), North Carolina (NC) , South Carolina (SC), Virginia (VA), West Virginia (WV), Arkansas (AR), Louisiana (LA), Oklahoma (OK), Texas (TX); Illinois (IL0, Indiana (IN), Michigan (MI), Ohio (OH), Wisconsin (WI), Iowa (IA), Kansas (KS), Missouri (MO), Nebraska (NE), North Dakota (ND), South Dakota (SD); Arizona (AZ), Colorado (CO), Idaho (ID), Montana (MT), Nevada (NV), New Mexico (NM), Utah (UT), Wyoming (WY), Alaska (AK), California (CA), Oregon (OR), Washington (WA).
